# Etoricoxib‐induced pleural effusion: A case report

**DOI:** 10.1002/ccr3.8247

**Published:** 2023-11-20

**Authors:** Bishal Kumar Sah, Ravi Ranjan Pradhan, Sangam Shah, Bishal Gaurav

**Affiliations:** ^1^ Institute of Medicine Tribhuvan University Maharajgunj Nepal; ^2^ Department of Internal Medicine Madhesh Institute of Health Sciences Janakpurdham Nepal; ^3^ Maharajgunj Medical Campus, Institute of Medicine Tribhuvan University Maharajgunj Nepal

**Keywords:** etoricoxib, Nepal, pleural effusion

## Abstract

**Key Clinical Message:**

Drug‐induced pleural effusion is extremely rare. It is the diagnosis of exclusion. This condition can be suspected if the patient has been exposed to a likely causative drug, develops new signs and symptoms, and has a remittance of these symptoms once the drug is withheld.

**Abstract:**

Etoricoxib has been a very popular nonsteroidal anti‐inflammatory drug and is prescribed widely due to its fewer gastrointestinal side effects. Pleural effusion caused by etoricoxib is rarest among the side effects. Here, we report a case of a 45‐year‐old female with pleural effusion induced by etoricoxib.

## Graphical abstract

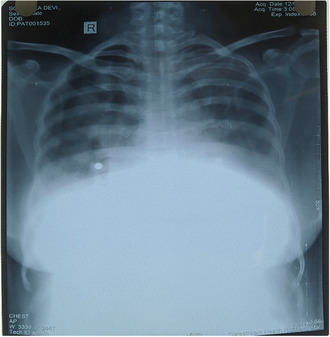

## 1 INTRODUCTION

The drug‐induced pleural disease might be localized to the pleura, accompany the parenchymal disease, or occur as a component of a bigger, potentially fatal systemic reaction. The pleura can be affected by several regularly used medications. However, pleural effusions are by far the most common symptoms. Several assumptions explain the underlying pathogenetic mechanisms, including hypersensitivity reactions, oxidative stress in mesothelial cells, dose‐dependent toxic effect, fluid retention, and chemical inflammation induction.^1^ There are two types of drugs linked to pleural effusion. Warfarin, diltiazem, simvastatin, imidapril, propylthiouracil, and mesalamine are among the medications in the first group, which are extremely heterogeneous.^2^ Nitrofurantoin, daptomycin, and tosufloxacin are among the chemotherapeutics and antibiotics in the second group. Etoricoxib, a selective COX‐2 inhibitor, has recently been found to cause pleural effusion. Here, we report a case of a 45‐year‐old female who developed pleural effusion after 2 weeks of etoricoxib intake. To our knowledge, this is the second case reported till now.

## 2 CASE PRESENTATION

A 45‐year‐old female presented to our center with chief complaints of shortness of breath for last 8 days. Initially, she had exertional dyspnea that developed while doing regular activities (New York Heart Association [NYHA II]). Later, she developed shortness of breath developed even at rest (NYHA‐IV). She had a 2‐week history of shoulder pain and movement restriction, for which she was diagnosed with frozen shoulder and treated with etoricoxib 60 mg and pantoprazole 40 mg daily.

On examination, she was well‐oriented to time, place, and person. On general physical examination, she had no pallor, icterus, cyanosis, dehydration, or clubbing. Her blood pressure was 102/78 mmHg, pulse rate was 80 beats/min, and respiratory rate was 21 breaths/min. There was decrease in the entry of air on both sides. The rest of the physical examinations were unremarkable.

**FIGURE 1:**
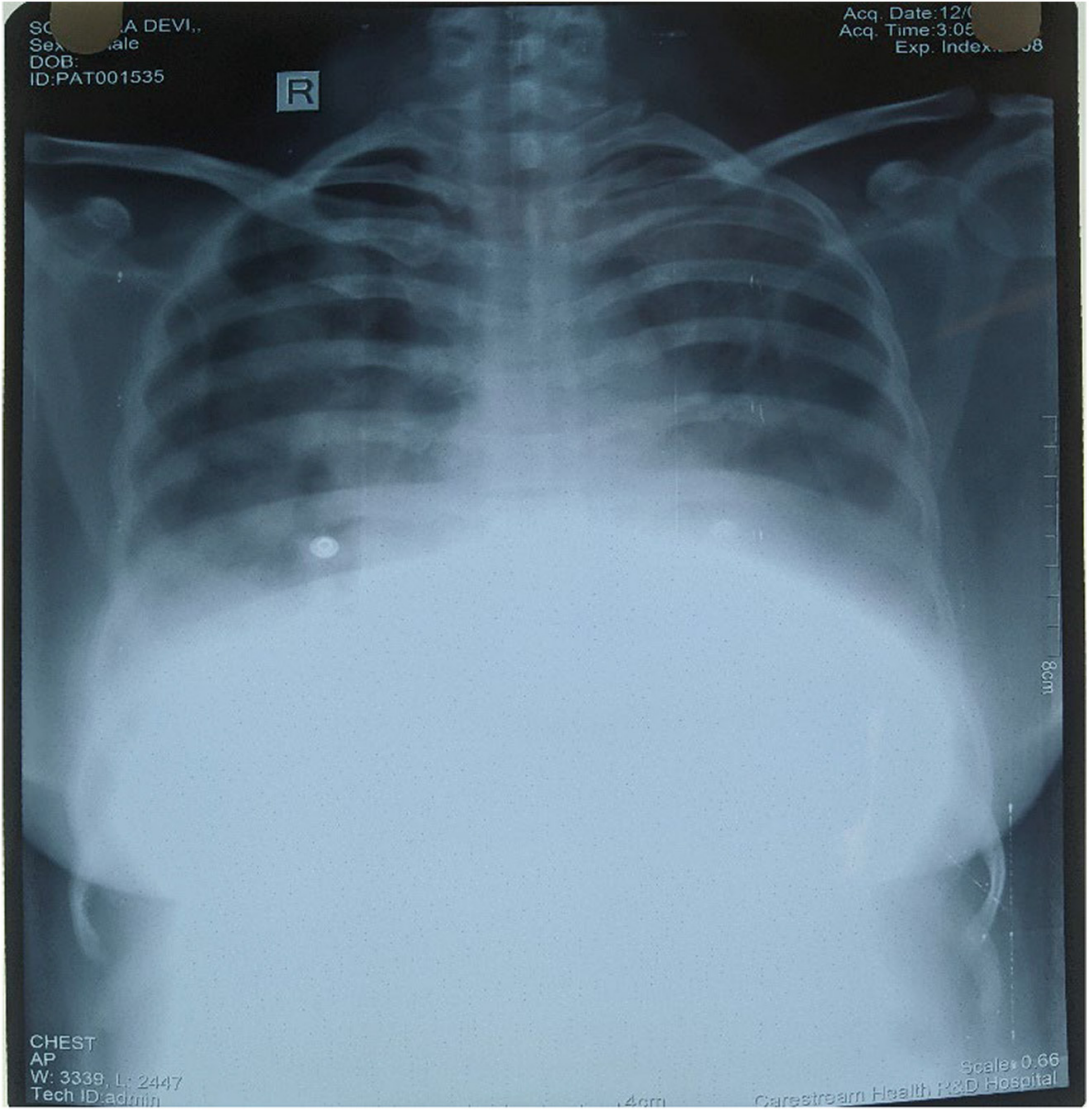
Chest X‐ray PA view showing bilateral pleural effusion.

Her blood count was normal including the eosinophil and platelet count. Her renal and liver function test was normal. The details of the laboratory investigation are shown in Table 1. Chest X‐ray showed bilateral pleural effusion (Figure 1). Her erythrocyte sedimentation rate and C‐reactive protein were normal, the tuberculin test was negative, and serum amylase was normal. Her fasting and postprandial blood sugar was normal. Pleural fluid analysis revealed a transudative type of pleural effusion, without any abnormal cytology with adenosine deaminase level within normal limits for tuberculosis. The ultrasound scan confirmed the presence of bilateral mild pleural fluid collection. A plain X‐ray of the spine did not show any abnormality. Electrocardiogram and echo were normal.

**TABLE 1:** Lab parameters of the included patient.

Lab parameters	Values
Hemoglobin	11.4 g %
Total leucocyte count	85,900 cells/mm^3^
Neutrophils	54.3%
Lymphocytes	32.4%
Platelets	170,000 cells/mm^3^
Random blood sugar	108 mg/dL
Sodium	142.7 mmol/L
Potassium	4.68 mmol/L
Urea	40 mg/dL
Creatinine	1.3 mg/dL
Total protein	6.9 g/dL
Total bilirubin	0.8 mg/dL
Direct bilirubin	0.3 mg/dL
Alanine aminotransferase (SGPT)	40.8 U/L
Aspartate aminotransferase (SGOT)	31.2 U/L

She was diagnosed to have bilateral pleural effusion and was managed conservatively. Etoricoxib was suspected of causing pleural effusion; thus, she was taken off the medication. She improved after withdrawal from the drug and was discharged on oral medications. On follow‐up, after 2 weeks of discharge, she had no fresh issues.

## 3 DISCUSSION

Etoricoxib is a COX‐2 inhibitor with a high level of enzyme selectivity. In the treatment of patients with arthritis and other painful illnesses, it is an alternative to various selective and classic nonsteroid anti‐inflammatory drugs. Etoricoxib is contraindicated in people with uncontrolled hypertension, ischemic heart disease, or stroke, and it should be used with caution in patients with heart disease risk factors, according to the European Medicines Agency.^3^ When looking at etoricoxib's side effects, it is clear that the drug rarely causes pleural effusion, and there are only a few published case reports.^2^

The onset of dyspnea in our patient coincides with the start of etoricoxib therapy, with no additional etiologies identified by a thorough study that ruled out all common illnesses, strengthening our conclusion. As shown in our patient, the symptoms could be severe enough to produce severe dyspnea and necessitate hospitalization. Eosinophilia of the pleural fluid is usually associated with drug‐induced effusions, although it was not present in our patient.

Symptoms may arise immediately after the first dose or later, with a latent period of weeks to months as with any other drug‐related pleural illness.^4^ Although the resolution of pleural effusion following medication withdrawal and reoccurrence after drug readministration are critical in determining causality, rechallenging the patient with the suspected drug is ethically objectionable in most cases, including this one. Causality assessment of etoricoxib‐induced pleural effusion was done using the Naranjo algorithm and revealed a score of 7, which is classified as probable (Table 2).^4^

**TABLE 2:** Causality assessment of etoricoxib‐induced pleural effusion using the Naranjo algorithm.

Question	Yes	No	Do not know	Score
1. Are there previous conclusive reports on this reaction?	+1	0	0	+1
2. Did the adverse event appear after the suspected drug was administered?	+2	−1	0	+2
3. Did the adverse event improve when the drug was discontinued or a specific antagonist was administered?	+1	0	0	+1
4. Did the adverse event reappear when the drug was readministered?	+2	−1	0	0
5. Are there alternative causes that could on their own have caused the reaction?	−1	+2	0	+2
6. Did the reaction reappear when a placebo was given?	−1	+1	0	0
7. Was the drug detected in blood or other fluids in concentrations known to be toxic?	+1	0	0	0
8. Was the reaction more severe when the dose was increased or less severe when the dose was decreased?	+1	0	0	0
9. Did the patient have a similar reaction to the same or similar drugs in any previous exposure?	+1	0	0	0
10. Was the adverse event confirmed by any objective evidence?	+1	0	0	+1
Total score				7

A comprehensive drug history should alert the treating physician to the possibility of a drug‐related process, including the date of drug exposure and symptom start. Eosinophilia in the pleural fluid could help the case. Eosinophilic pleural effusion has been linked to medications such as nitrofurantoin, dantrolene, bromocriptine, valproic acid, isotretinoin, propylthiouracil, and angiotensin‐converting enzyme inhibitors. Unless therapeutic aspiration is required to treat the symptoms, pleural effusion usually clears itself when the drug is stopped.

## 4 CONCLUSION

Based on our experience with this case, we recommend that doctors review the pharmaceuticals the patient has taken or is taking while dealing with pleural effusions, and if any are found, the medication should be discontinued promptly. Even though etoricoxib has lower gastrointestinal toxicity and better tolerance than other common medications when used for long period, clinicians should be wary of potential cardiovascular hazards and avoid using it in patients with cardiovascular risk factors.

## AUTHOR CONTRIBUTIONS

**Bishal Kumar Sah:** Conceptualization; writing – original draft; writing – review and editing. **Ravi Ranjan Pradhan:** Writing – original draft; writing – review and editing. **Sangam Shah:** Writing – original draft; writing – review and editing. **Bishal Gaurav:** Writing – original draft; writing – review and editing.

## FUNDING INFORMATION

None.

## CONFLICT OF INTEREST STATEMENT

There are no conflicts of interest to declare.

## ETHICS STATEMENT

None.

## CONSENT

Written informed consent was obtained from the patient to publish this report in accordance with the journal's patient consent policy.

## Data Availability

All the required information is in the manuscript itself.

## References

[1] Antony VB . Drug‐induced pleural disease. Clin Chest Med. 1998;19(2):331‐340. 9646984 10.1016/s0272-5231(05)70080-0

[2] Balasingam N , Thirunavukarasu K , Selvaratnam G . Etoricoxib‐induced pleural effusion: a case for rational use of analgesics. J Pharmacol Pharmacother. 2015;6:231‐232. 26816478 10.4103/0976-500X.171876 PMC4714395

[3] Fanelli A , Ghisi D , Aprile PL , Lapi F . Cardiovascular and cerebrovascular risk with nonsteroidal anti‐inflammatory drugs and cyclooxygenase 2 inhibitors: latest evidence and clinical implications. Ther Adv Drug Saf. 2017;8(6):173. 28607667 10.1177/2042098617690485 PMC5455842

[4] Adverse Drug Reaction Probability Scale (Naranjo) in DRUG INDUCED LIVER Injury [Internet]. In: LiverTox: Clinical and Research Information on Drug‐Induced Liver Injury. National Institute of Diabetes and Digestive and Kidney Diseases; 2012. Accessed June 27, 2021. http://www.ncbi.nlm.nih.gov/pubmed/31689026 31689026

